# The Doctor’s Difficulty

**Published:** 1873-01

**Authors:** 


THE DOCTOR'S DIFFICULTIES.—Some of these are well stated in the following extract from the *New York Tribune*:—

Dr. Wheeler, of Dover, N. H., at Dartmouth College, addressing the graduating medical class, began by remarking that "the science of medicine has been, and is now, a growth, and, consequently, has not yet reached perfection;" a sensible observation. The main trouble with medicine is, that man was born to die, not merely of old age, but of various diseases, at various stages of life. Recovery from an illness depends upon several conditions, with some of which the medical man who is called in, has nothing to do. He may be sent for too tardily; his advice may not be followed, and his prescriptions may be negligently dispensed, or altogether dispensed with. He cannot keep watch and ward by every bedside, to prevent nurses from dosing their victims into the grave. And, more than all, however much he may know of theory and practice, there will remain a great many things of which he is ignorant, while patients and their friends distract him, and cry unto him, in the same breath, for succor.

